# Transcriptomic analysis reveals the potential crosstalk genes and immune relationship between IgA nephropathy and periodontitis

**DOI:** 10.3389/fimmu.2023.1062590

**Published:** 2023-01-30

**Authors:** Xiaoli Gao, Ziyi Guo, Pengcheng Wang, Zhiqiang Liu, Zuomin Wang

**Affiliations:** Department of Stomatology, Beijing Chaoyang Hospital, Capital Medical University, Beijing, China

**Keywords:** periodontitis, IgA nephropathy, transcriptomic analysis, crosstalk genes, immune infiltration

## Abstract

**Background:**

It is well known that periodontitis has an important impact on systemic diseases. The aim of this study was to investigate potential crosstalk genes, pathways and immune cells between periodontitis and IgA nephropathy (IgAN).

**Methods:**

We downloaded periodontitis and IgAN data from the Gene Expression Omnibus (GEO) database. Differential expression analysis and weighted gene co-expression network analysis (WGCNA) were used to identify shared genes. Then, Gene Ontology (GO) and Kyoto Encyclopedia of Genes and Genomes (KEGG) pathway enrichment analyses were performed on the shared genes. Hub genes were further screened using least absolute shrinkage and selection operator (LASSO) regression, and a receiver operating characteristic (ROC) curve was drawn according to the screening results. Finally, single-sample GSEA (ssGSEA) was used to analyze the infiltration level of 28 immune cells in the expression profile and its relationship with shared hub genes.

**Results:**

By taking the intersection of WGCNA important module genes and DEGs, we found that the *SPAG4, CCDC69, KRT10, CXCL12, HPGD, CLDN20* and *CCL187* genes were the most important cross-talk genes between periodontitis and IgAN. GO analysis showed that the shard genes were most significantly enriched in kinase regulator activity. The LASSO analysis results showed that two overlapping genes (*CCDC69* and *CXCL12*) were the optimal shared diagnostic biomarkers for periodontitis and IgAN. The immune infiltration results revealed that T cells and B cells play an important role in the pathogenesis of periodontitis and IgAN.

**Conclusion:**

This study is the first to use bioinformatics tools to explore the close genetic relationship between periodontitis and IgAN. The *SPAG4, CCDC69, KRT10, CXCL12, HPGD, CLDN20* and *CCL187* genes were the most important cross-talk genes between periodontitis and IgAN. T-cell and B-cell-driven immune responses may play an important role in the association between periodontitis and IgAN.

## Introduction

Periodontitis is a multifactorial chronic inflammatory disease associated with dysbiosis of the oral microbiota, and approximately 50% of adults suffer from this chronic disease (1), which is characterized by gingival inflammation and irreversible destruction of periodontal attachments (i.e., cementum, periodontal ligament, and alveolar bone). Recent advances suggest that periodontal disease initiation and propagation occur through dysbiosis of the commensal oral microbiota (dental plaque), which then interacts with the immune defenses of the host, leading to inflammation and disease (2).

In recent decades, there has been interest in the possibility that the presence of periodontal disease may be a causative or aggravating factor in some systemic diseases (3). Three mechanisms currently support a link between periodontitis and systemic disease: metastatic infections, dissemination of bacterial toxins and immunological injury (3). The first two mechanisms suggest the transmission of bacteria and their bacterial products from the oral cavity to the systemic level. The third theory suggests that systemic damage is the result of an inflammatory cascade response that begins in the oral cavity.

IgA nephropathy (IgAN) is the most prevalent glomerular disease worldwide and is associated with a poor prognosis (4). The pathogenesis of IgAN is complex and may involve a variety of different pathways (5). Studies indicate that infection may play a major role in the pathogenesis of IgAN (6). It is well known that upper respiratory tract infections often worsen IgAN, particularly acute tonsils. IgAN is thought to be a tonsillion-related disease caused by a breakdown in the immune tolerance of the resident bacteria in the tonsil (7), and several bacterial species have been reported as antigens in the pathogenesis of IgAN (8–10). Bacteria involved in chronic oral infections may be pathogenic candidates. Negasawa el. indicated that *P. gingivalis* (the main pathogenic bacteria of periodontitis) in tonsils was detected with significantly higher prevalence in IgAN patients than in habitual tonsillitis patients (11). Moreover, Misaki el al suggested that harboring *C. rectus* in the oral cavity could be associated with proteinuria in IgAN patients (12). The complete elimination of oral infection lesions can optimize the treatment effect of tonsillectomy plus methylprednisolone (MP) pulse therapy and promote the recovery of IgAN (13).

At present, the relationship between periodontitis and IgAN requires further investigation, especially in terms of cellular and molecular mechanisms. With the rapid development of microarray and high-throughput sequencing technology, bioinformatics techniques are often used to study the crosstalk between diseases. In this study, we used bioinformatics methods to explore the potential crosstalk genes between periodontitis and IgAN and evaluated the interaction between these potential crosstalk genes and infiltrating immune cells to gain a deeper understanding of the pathophysiological processes that may link periodontitis and IgAN.

## Materials and methods

### Data download

We obtained the gene expression of periodontitis and IgAN, which was downloaded from the GEO database. Datasets are available at https://www.ncbi.nlm.nih.gov/geo/ for more information. Based on the GPL570-55599 platform, the GSE16134 dataset contains 310 gingival papillae from 120 subjects undergoing periodontal surgery (241 “diseased”, 69 “healthy”). To estimate the diagnostic efficiency, the GSE10334 dataset based on GPL570-55599 was downloaded, containing 247 gingival papillae (183 “diseased” and 64 “healthy”) from 90 patients with periodontitis. Tissue samples were taken from patients with moderate to severe periodontitis and the criteria for selection of the samples were: “Diseased” sites showed bleeding on probing (BoP), had interproximal probing depth (PD) ≥ 4 mm, and concomitant attachment loss (AL) ≥ 3 mm; “Healthy” sites showed no BoP, had PD ≤ 4 mm and AL ≤ 2 mm.

Gene expression datasets investigating IgAN (GSE93798) were based on the GPL22945 platform and included 42 samples (20 IgAN patients and 22 healthy controls). Samples were taken from routine kidney biopsies, and biopsies from patients diagnosed with IgAN were singled out for further experiments, biopsies from healthy living kidney transplant donors were used as controls. To estimate the diagnostic efficiency, we also downloaded GSE73953 (based on the GPL4133 platform), containing 15 IgAN samples and 2 healthy controls. Those patients had been comprehensively diagnosed by kidney biopsy, medical history, and blood examinations. The detailed information for the samples included in this study is summarized in **Supplementary file 1**.

### Identification of DEGs

The original expression matrix was normalized and processed using R (4.0.4) software. The “limma” R package was used to screen the differentially expressed genes (DEGs) from the GSE16134 and GSE93798 datasets. The DEGs were screened for GSE16134 with an adjusted *P* value < 0.05 and |log FC| ≥ 0.9, and the DEGs were screened for GSE93798 with an adjusted *P* value < 0.05 and |log FC| ≥ 1. R software was used to draw a differential gene clustering heatmap and volcano map.

### WGCNA network construction and module identification

WGCNA is a bioinformatics analysis method used to describe gene association patterns among different samples. It can cluster genes with similar expression patterns and analyse the association between modules and specific traits or phenotypes (14). The WGCNA R software package was used to construct the co-expression network. Genes with an adjusted *P* value < 0.05 were included in the WGCNA. First, hierarchical clustering was performed using the standard R function “Hculst” to assess whether there were any obvious outliers. Second, to make the gene expression relationship conform to the scale-free network, the “pickSoftThreshold” function was used to select the appropriate soft thresholding power β. Third, the “adjacency” function was used to convert the gene expression similarity matrix into an adjacency matrix based on the soft-thresholding parameter β. Fourth, the adjacency matrix obtained in the previous step was transformed into a topological overlap matrix (TOM) to minimize the effects of noise and spurious associations. Finally, hierarchical clustering and the dynamic tree cut function were used to detect modules, and Pearson correlation was used to investigate the correlation between modules and clinical characteristics of patients (*P* < 0.05).

### Identification of shared genes and pathway enrichment

A combined analysis of the genes screened by WGCNA and DEGs was conducted by drawing Venn diagrams. Overlapping genes were considered core shared genes and were extracted for further functional enrichment analysis. Gene Ontology (GO) and Kyoto Encyclopedia of Genes and Genomes (KEGG) pathway enrichment analyses were performed using the “enrichplot” and “ggplot2” packages in R. Statistical significance was set at *P* < 0.05.

### Feature selection by the least absolute shrinkage and selection operator

Lasso is a popular method for regression that uses an ℓ1 penalty to achieve a sparse solution (15). We used the “glmnet” language package in R to perform least absolute shrinkage and selection operator (LASSO) regression to screen the best predictors of periodontitis and IgAN in the above DEGs and intersection of WCGNA.

### Candidate biomarker expression levels and diagnostic value

The R software ggplot2 package boxplots were used to assess the expression levels of the hub genes (*P* < 0.05). The area under the curve (AUC) of receiver operating characteristic (ROC) was utilized to determine the effectiveness of potential biomarkers on the datasets (GSE16134, GSE93798, GSE10334 and GSE73963) using the pROC R package.

### ssGSEA

ssGSEA was performed by the “GSVA” R package to analyse the infiltration of 28 immune cells in diseased and normal samples. To investigate the correlation between *core genes* and the abundances of infiltrating immune cells, *p* values were calculated based on Spearman’s rank correlation tests (*P* < 0.05).

## Results

### Identification of DEGs

**Figure 1:**
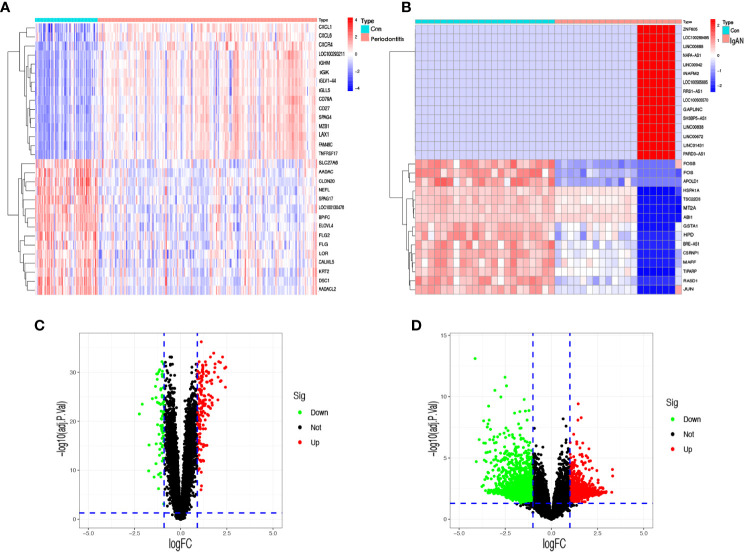
Identification of differentially expressed genes. **(A)** A heatmap of the top 30 DEGs in GSE16134. **(B)** A heatmap of the top 30 DEGs in GSE93798. **(C)** A volcano plot of DEGs in GSE16134. **(D)** A volcano plot of DEGs in GSE93798. Con: control; IgAN: IgA nephropathy.

**Figure 2:**
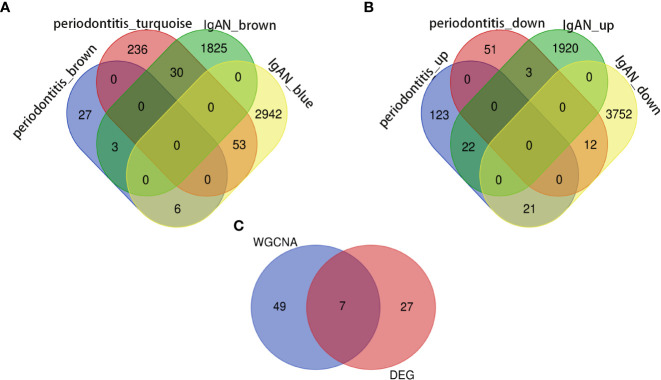
Identification of the shared genes. **(A)** Venn diagram shows that 56 genes overlap in the IgAN and periodontitis modules. **(B)** Venn diagram showing an overlap of 34 DEGs between IgAN and periodontitis. **(C)** Venn diagram showing that seven core genes were crossed and overlapped between the genes screened by WGCNA and DEGs. IgAN: IgA nephropathy; DEG: differentially expressed gene; WGCNA: weighted gene co-expression network analysis.

In the periodontitis dataset GSE16134, a total of 232 DEGs, consisting of 166 upregulated DEGs and 66 downregulated DEGs, were identified. In the IgAN dataset GSE93798, a total of 5,730 DEGs, consisting of 1,945 upregulated DEGs and 3,785 downregulated DEGs, were identified. The heatmaps (**Figures 1A, B**) demonstrated the top 15 DEGs of the two diseases, and the volcano maps (**Figures 1C, D**) were used to show the expression pattern of DEGs in both diseases. There were 34 overlapping differentially expressed genes between periodontitis and IgAN (**Figure 2B**).

### WGCNA network construction and module identification

**Figure 3:**
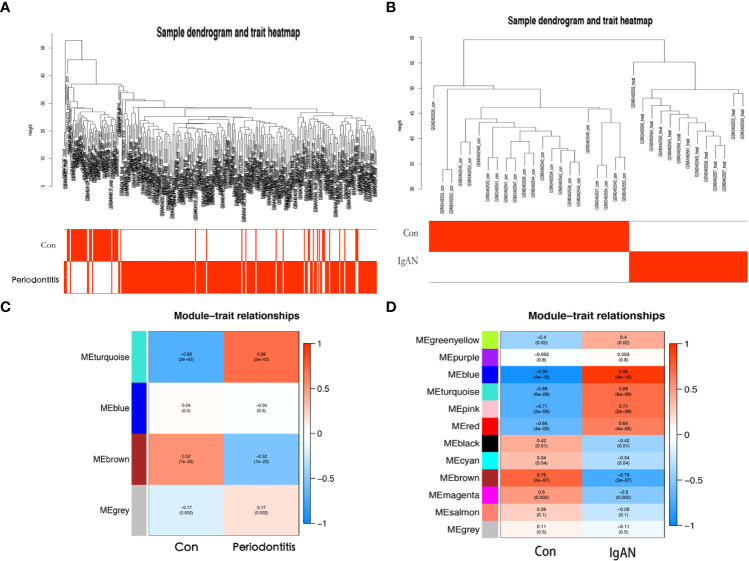
Coexpression analysis for differentially expressed genes. **(A)** Sample dendrogram and trait heatmap in GSE16134. **(B)** Sample dendrogram and trait heatmap in GSE93798. **(C)** Heatmap of the module-trait relationships in GSE16134. **(D)** Heatmap of the module-trait relationships in GSE16134. Con: control; IgAN: IgA nephropathy.

Outliers were checked by sample clustering, and no samples were removed in either GSE16134 or GSE93798 (**Figures 3A, B**). To ensure a scale-free network, we calculated the scale-free fit index and mean connectivity. The power of β = 6 was chosen for the soft thresholding for GSE16134, and the *β* value was 13 for GSE93798. We obtained 4 modules in the co-expression network constructed by periodontitis samples and 12 modules in the network constructed by IgAN samples (**Figures 3B, C**). To identify genes associated with the progression of diseases, we analysed the association between modules and clinical phenotypes. For periodontitis, the turquoise module had the strongest positive relation (r = 0.68, *P* < 0.001), while the brown module had the strongest negative relation (r = -0.52, *P* < 0.001) in the GSE16134 database. For IgAN, the blue module showed the strongest positive correlation (r = 0.95, P < 0.001), and the brown module had the strongest negative correlation (r = -0.75, P < 0.001) in the GSE93798 database. The intersection of the periodontitis and IgAN hub modules was drawn by a Venn diagram, and 56 intersection genes were obtained (**Figure 2A**).

### Identification of shared genes and pathway enrichment

**Figure 4:**
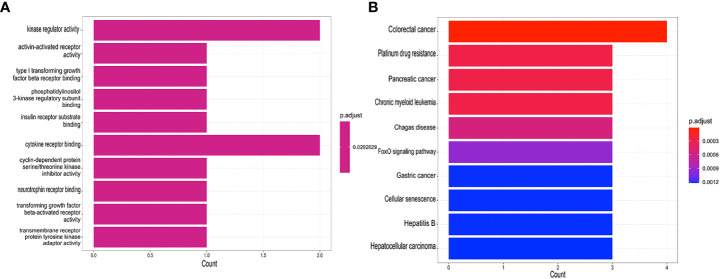
Functional enrichment analyses of the shared genes. **(A)** GO analysis of the shared genes. **(B)** KEGG pathway enrichment analysis of the shared genes.

Seven core genes (*SPAG4, CCDC69, KRT10, CXCL12, HPGD, CLDN20, CCL18)* were crossed and overlapped between the genes screened by WGCNA and DEGs, which are potential crosstalk genes between both diseases (**Figure 2C**). GO and KEGG enrichment analyses were performed on the above 7 genes to explore the common regulatory pathways. The GO analysis showed that the shared genes might be related to kinase regulator activity, activin-activated receptor activity and type I transforming growth factor beta receptor binding (**Figure 4A**). The KEGG analysis showed that these genes might be mainly involved in colorectal cancer, platinum drug resistance, and pancreatic cancer (**Figure 4B**).

### Potential shared diagnostic genes selection by least absolute shrinkage and selection operator

**Figure 5:**
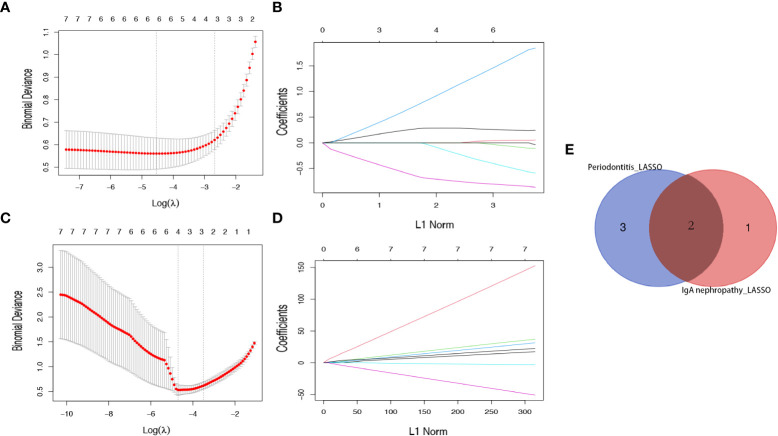
Identification of *potential shared diagnostic genes* by the LASSO regression model. **(A)** Tenfold cross-validation to select the optimal tuning parameter log (lambda) in the GSE16134 database. **(B)** Tenfold cross-validation to select the optimal tuning parameter log (lambda) in the GSE93798 database. **(C)** LASSO coefficient profiles of diagnostic genes in the GSE16134 database. **(D)** LASSO coefficient profiles of diagnostic genes in the GSE93798 database. **(E)** Venn diagram showing the optimal diagnostic biomarkers.

Subsequently, a LASSO regression algorithm was performed to identify the potential shared diagnostic genes. In GSE16134, LASSO analysis identified six out of the seven core cross-genes under the most appropriate λ=0.011 (**Figures 5A, C**). In GSE93798, LASSO analysis identified four out of the seven core cross-genes under the most appropriate λ=0.011 (**Figures 5B, D**). Finally, two overlapping genes (*CCDC69* and *CXCL12*) were discovered to be the optimal shared diagnostic biomarkers for periodontitis and IgAN (**Figure 5E**).

### Candidate biomarker expression levels and diagnostic value

**Figure 6:**
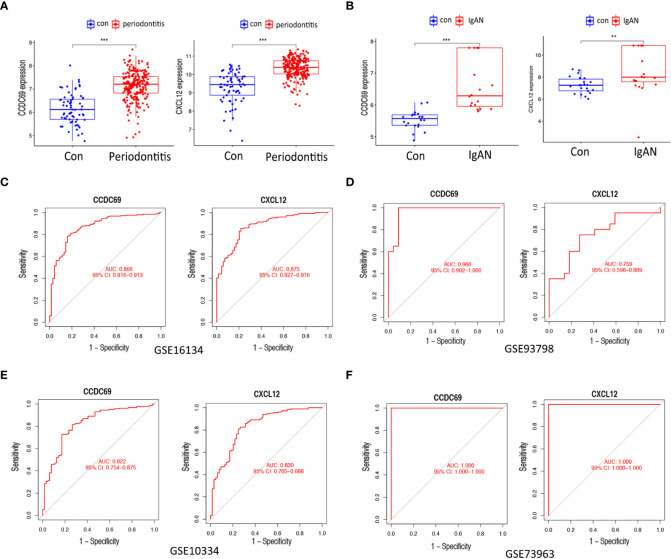
Expression pattern validation and diagnostic value. **(A)** Expression of CXCL12 and CCDC69 in GSE16134. **(B)** Expression of CXCL12 and CCDC69 in GSE93798. **(C)** ROC curve of the shared diagnostic genes in GSE16134. **(D)** ROC curve of the shared diagnostic genes in GSE93798. **(E)** ROC curve of the shared diagnostic genes in GSE10334. **(F)** ROC curve of the shared diagnostic genes in GSE73953. Con: control; IgAN: IgA nephropathy. *P* < 0.05; ^**^ *P* < 0.01; ^***^ *P* < 0.001.

**Figures 6A, B** show the expression levels of the two candidate biomarkers. Both *CCDC69* and *CXCL12* were upregulated in periodontitis and IgAN. Furthermore, we evaluated the sensitivity and specificity of candidate biomarkers. In the GSE16134 dataset (**Figure 6C**), these two biomarkers had good diagnostic value: *CCDC68* (AUC = 0.866) and *CXCL12* (AUC = 0.875). In the GSE93798 dataset (**Figure 6D**), *CXCL12* had a higher diagnostic value for IgAN (AUC = 0.759), while *CCDC68* had a near perfect diagnostic value (AUC = 0.966). We then performed external validation for the diagnostic efficacy of *CCDC69* and *CXCL12* in the periodontitis dataset GSE10034 and the IgAN dataset GSE73963, and all showed potent predictive performance (**Figures 6E, F**).

### Immune cell infiltration and its correlation with candidate biomarkers

**Figure 7:**
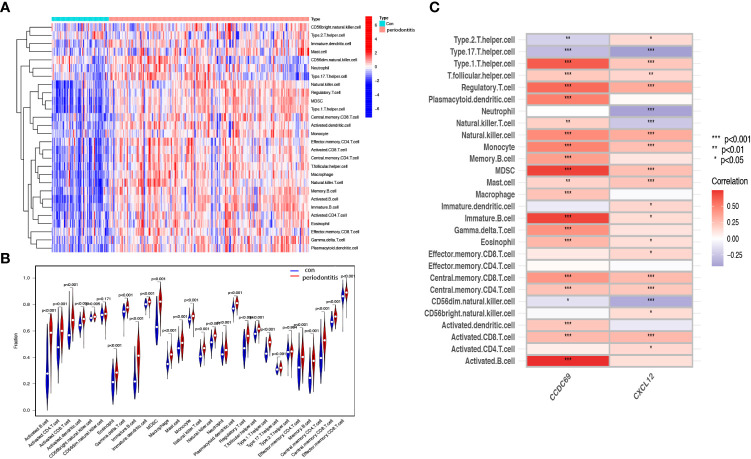
Analysis of immune infiltration associated with periodontitis. Heat **(A)** and violin plot **(B)** showing the distribution of 28 immune cells in the GSE16134 sample. **(C)** The relationship between diagnostic genes and immune cell infiltration. Con, control.

**Figure 8:**
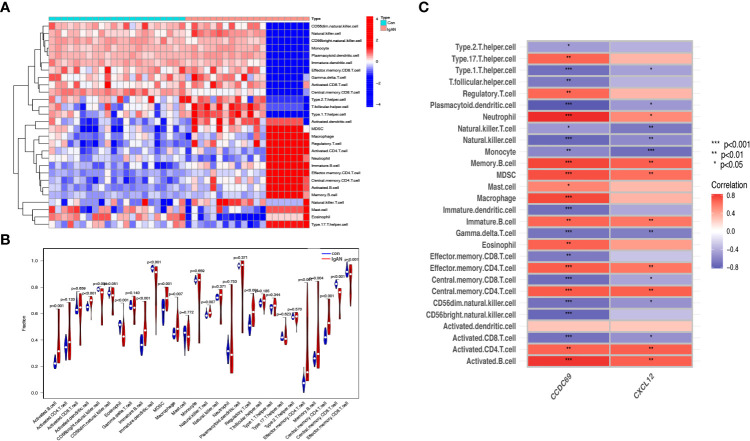
Analysis of immune infiltration associated with IgAN. Heat **(A)** and violin plot **(B)** showing the distribution of 28 immune cells in the GSE93798 sample. **(C)** The relationship between diagnostic genes and immune cell infiltration. Con, control; IgAN, IgA nephropathy.

We further investigated the difference in immune cell infiltration in different samples. Twenty-eight types of immune cells in the GSE10334 samples were identified and are presented in the heatmap and violin plot (**Figures 7A, B**). **Figures 8A, B** show the distribution of 28 immune cells in the GSE93798 sample. We found that the infiltration of activated B cells, activated dendritic cells, immature B cells, MDSCs, macrophages, natural killer T cells, regulatory T cells, effector memory CD4 T cells, memory B cells, and central memory CD4^+^ T cells increased more significantly in both periodontitis and IgAN samples. In addition, correlation analysis of immune cells with candidate biomarkers demonstrated that central memory CD4^+^ T cells, MDSCs and immature B cells were positively correlated with *CCDC69* and CXCL12 in periodontitis samples and IgAN samples (**Figures 7C**, **8C**).

## Discussion

In this study, we integrated the transcriptomes of periodontitis and IgAN and used WGCNA for the first time to explore the common mechanism between the two, revealing potential crosstalk genes, shared pathways, and associated immune cells. By taking the intersection of WGCNA important module genes and DEGs, we found that the *SPAG4, CCDC69, KRT10, CXCL12, HPGD, CLDN20* and *CCL187* genes were the most important cross-talk genes between periodontitis and IgAN, and these genes may be related to kinase regulator activity. Finally, *CXCL12* and *CCDC69* were identified as diagnostic markers with good value. The immune infiltration results revealed that T cells and B cells play an important role both in the pathogenesis of periodontitis and IgAN.

The results of this study suggest that the core cross-talk genes between periodontitis and IgAN are associated with kinase regulator activity. Kinases are enzymes that catalyze the transfer of phosphate groups, usually from ATP to substrate molecules. Periodontal pathogens play a critical role in the development and progression of periodontitis. *Porphyromonas gingivalis* is a keystone pathogen of periodontitis, whose main virulence factors include gingipain proteases and peptidylarginine deiminase (PPAD), and the phosphorylation of virulence factors is crucial for their processing and secretion (16). In addition, *α-hemolytic streptococcus*, as one of the main bacterial groups in the oral cavity, can promote the expression of homeodomain-interacting protein kinase 2 (HIPK2) in tonsil lymphocytes, which may mediate IgA1 glycosylation (17). Thus, periodontal or oral pathogens induced kinase regulator activity may play an important role in the common pathophysiology of the two diseases.

In this study, the possible immune relationship between IgAN and periodontitis was preliminarily explored, and it was found that the immune pattern was significantly different in the periodontitis and IgAN groups compared to the control group, and B and T cells increased more significantly in both periodontitis and IgAN samples. Primary IgAN is characterized by IgA deposition in the mesangial region of the glomerulus (18). The subclass of IgA in this deposition is IgA1, which may be produced in the mucosal region of the upper respiratory tract, including the tonsils or mucosal tissues in the oral region (19). The tonsil parenchyma consists of lymphoid follicles dominated by B cells and T-cell-dependent interfollicular regions (20, 21). Tonsillar focal infection may elevate the generation of CD8 T cells and the aberration of immunoglobulin production (22). Recent studies have suggested that chronic oral bacterial infections, especially red complexes of periodontopathic bacterial species, may be involved in the pathogenesis of IgAN (11).A recent review article highlighted that in the crypt of the tonsil, macrophages/dendritic cells engulf antigens, such as oral bacteria, and present them to helper T cells; activated helper T cells stimulate and activate mature B cells in the tonsillar germinal center to produce Gd-IgA1, and then the produced Gd-IgA1 enters the systemic circulation through the lymphatic system and causes kidney injury (23).In addition to tonsils, chronic infection during periodontitis may also stimulate mature B-cell proliferation in mesenteric lymph nodes and lead to increased IgA secretion through mucosal immune response, thus promoting the occurrence and progression of IgAN (24). Furthermore, T cells and their cytokines are involved in posttranslational modification of IgA1 hinge region (25). Some studies have found that the periodontal pathogen *porphyromonas gingivalis* can alter T cell response (26). Therefore, we speculated that periodontal infection might lead to abnormal IgA deposition through activation of T - and B-cell-mediated mucosal immunity.

In order to minimize the influence of overfitting and improve the quality of performance indicators, as many samples as possible should be selected for clinical biomarker discovery experiments (27). In our study, the GSE16134 dataset included a total of 198 gingival tissue samples and the GSE93798 dataset included 42 kidney tissue samples. The performance of a biomarker is often assessed using the area under the receiver operator curve (AUC). The value of AUC is between 0 and 1, and the higher the value, the better the overall performance of the test (28). In this study, ROC analysis found that the AUC of CXCL12 was 0.875 for predicting periodontitis and 0.759 for predicting IgAN, and the AUC of CCDC69 was 0.866 for predicting periodontitis and 0.966 for predicting IgAN. Therefore, both CXCL12 and CCDC69 have good predictive properties for periodontitis and IgAN.

As an important crosstalk gene between IgAN and periodontitis, *CXCL12* (CXC chemokine ligand 12) is a potent chemoattractant that belongs to the CXC chemokine family. Widely expressed in many tissues during development, it is a powerful chemoattractant of hematopoietic cells, which has been shown to promote their migration across the endothelial barrier (29). *CXCL12* levels increase during the development of periodontal disease and may recruit host defense cells to sites of inflammation, which may be involved in activating immune defense pathways during periodontal disease (30, 31). The following evidence suggests that it may be involved in the development and progression of IgAN mediated by periodontitis. It has been shown that GZMK CD8 memory T cells, mediated by *CXCL12*, have a prolonged residence time in the lytic region of neutrophils, which in turn can enhance antigen presentation (32). In some patients, *CXCL12* can directly trigger effector T cells and affect their own migratory activity by secreting IL-15, causing rejection (33). In an airway infection model established by *Pseudomonas aeruginosa*, *CXCL12* and *HMGB1* binding was found to strongly promote the activation of downstream immune cells (34). Decreased expression and activation levels of *CXCL12* were found in cyclosporin-immunosuppressed B cells as a mechanism for the treatment of autoimmune diseases by this class of drugs (35). Therefore, we assume that *CXCL12* may be involved in both pathologies by mediating processes such as post-infection T-cell activation in tonsils and mucosa, systemic migration of effector T cells, and secretion of B-cell-deficient IgA. In addition, it has been speculated that in IgAN patients, abnormal expression of chemokines and/or chemokine receptors causes IgA-producing cells destined for the secretory mucosal lamina propria to be mistakenly transferred to nonsecretory sites, such as the tonsil, bone marrow, and spleen (36). In IgAN, B cells may be involved in the production of galactose-deficient IgA1 (Gd-IgA1) and its antibodies (37), and the *CXCL12-CXCR4* axis is thought to be a major player in B-cell precursor homeostasis in the bone marrow (38). In summary, a potential role for chemokines in the interaction between periodontitis and IgAN seems likely, but further data are needed.

Our study found that *CCDC69* is also an important crosstalk gene between IgAN and periodontitis; however, there are currently few studies on *CCDC69*. Pal et al. noted that *CCDC69* acts as a scaffold to regulate the recruitment of midzone components and the assembly of the central spindle, which plays a crucial role in cell division (39). *CCDC69* is also involved in the immune response. The expression of *CCDC69* is positively correlated with immune cells (B cells, CD8+ cells, neutrophils, dendritic cells and lymphocytes) around the tumor, which can reflect the infiltration of immune cells (40). In this study, *CCDC69* and *CXCL12* had a significant positive correlation with central memory CD4^+^ T cells and immature B cells in both periodontitis samples and IgAN samples. This will highlight the common pathophysiology at the immune level and may be a key factor in understanding the relationship between periodontitis and IgAN.

Our study has several strengths. We first used the comprehensive and complex bioinformatics analysis as a new approach to understand the association between the two diseases. LASSO regression algorithm was performed to identify the potential shared diagnostic genes. Validation of external datasets improves the accuracy of predictions. There are also some limitations in this study. Our finding relied on different patient cohorts and were not validated in the same individual. A model of the combination of periodontitis and IgAN needs to be established to verify the potential relationship between the two diseases in the future. In addition, data on age, sex, medication, and comorbidities of patients in the samples were not considered in this study, which may be reliable for the current results.

## Conclusion

This study is the first to use bioinformatics tools to explore the close genetic relationship between periodontitis and IgA nephropathy. The *SPAG4, CCDC69, KRT10, CXCL12, HPGD, CLDN20* and *CCL187* genes were the most important cross-talk genes between periodontitis and IgAN. T-cell and B-cell-driven immune responses may play an important role in the association between periodontitis and IgAN.

## Data availability statement

The original contributions presented in the study are included in the article/**Supplementary Material**. Further inquiries can be directed to the corresponding authors.

## Author contributions

XG conceived of the procedure for the research and wrote the manuscript. ZG and PW helped analyse the study data. ZL and ZW revised the draft. All authors read and approved the final manuscript. All authors contributed to the article and approved the submitted version.
