# Supplementary material for: Transcriptomic analysis reveals the potential crosstalk genes and immune relationship between IgA nephropathy and periodontitis
Source: Front Immunol. 2023 Jan 30;14:1062590. doi: 10.3389/fimmu.2023.1062590 (PMC9924229; doi:10.3389/fimmu.2023.1062590)
Supplement: Supplementary file 1 [file Table_1.docx]

Supplementary Material

# Transcriptomic analysis reveals the potential crosstalk genes and immune relationship between IgA nephropathy and periodontitis

**Xiaoli Gao^*^, Ziyi Guo, Pengcheng Wang,** **Zhiqiang Liu** **†, Zuomin Wang** **†**

**†**Cocorresponding authors

Zuomin Wang, [wzuomin@sina.cn](mailto:wzuomin@sina.cn)

Zhiqiang Liu, [kokorora@sina.com](mailto:kokorora@sina.com)

Detailed information of included data

| **Disease** | **Series number** | **Platforms** | **Tissue** | **Case** | **Control** | **Total** | **Study period** |
| --- | --- | --- | --- | --- | --- | --- | --- |
| Periodontitis | GSE16134 | GPL570-55599 | Gingiva | 241 | 69 | 310 | Not mentioned in the original paper |
|  | GSE10334 | GPL570-55599 | Gingiva | 183 | 64 | 247 | between November 2004 and April 2007 |
| IgA nephropathy | GSE93798 | GPL22945 | Glomeruli | 20 | 22 | 42 | Not mentioned in the original paper |
|  | GSE73953 | GPL4133 | Peripheral blood mononuclear cells | 15 | 2 | 17 | From March 2008 to June 2008 |
